# The Clinical Significance and Risk Factors of Solitary Lymph Node Metastasis in Gastric Cancer

**DOI:** 10.1371/journal.pone.0114939

**Published:** 2015-01-29

**Authors:** Min Ma, Shi Chen, Bao-yan Zhu, Bai-Wei Zhao, Hua-She Wang, Jun Xiang, Xiao-Bin Wu, Yi-Jia Lin, Zhi-Wei Zhou, Jun-Sheng Peng, Ying-Bo Chen

**Affiliations:** 1 The State Key Laboratory of Oncology in South China, Cancer Center, Sun Yat-Sen University, Guangzhou, China; 2 Department of Gastric & pancreatic Surgery, Cancer Center, Sun Yat-Sen University, Guangzhou, China; 3 Department of Gastroenterology Surgery, The 6th Affiliated Hospital, Sun Yat-Sen University, Guangzhou, China; H. Lee Moffitt Cancer Center & Research Institute, UNITED STATES

## Abstract

**Aims:**

To assess the clinical significance and risk factors of solitary lymph node metastasis (SLM) in gastric carcinoma and establish a more accurate method to evaluate the possibility of lymph node metastasis (LM).

**Methods:**

A total of 385 patients with gastric carcinoma who underwent D2 lymphadenectomy at the Cancer Center of Sun Yat-Sen University were included in this research. Then we used a group of data from Sun Yat-sen University Gastrointestinal Hospital (SYSUGIH) to validate the accuracy of our developed method. The χ^2^ test, Kaplan–Meier analysis, log-rank test, COX model, and discriminate analysis were used to analyze the data with SPSS13.0.

**Results:**

We found that the LM number and pathological T staging were independent prognostic risk factors. CEA grading, LN status by CT, and T staging by CT were independent risk factors for LM in gastric carcinoma. In addition, we developed the equation *Y* = -5.0 + *X*_1_ + 1.8*X*_3_ + 0.7*X*_4_ (*X*_1_ = CEA grading, *X*_3_ = LN status by CT, *X*_4_ = T staging by CT) to evaluate the situation of LM. The data from SYSUGIH shows this equation has a better accuracy compared with CT.

**Conclusions:**

SLM is an independent risk factor in gastric cancer. And there was no survival difference between the skip metastasis group and the other SLM group (*P* = 0.659). It is inappropriate for the patient with SLM doing a standard D2 lymphadenectomy, due to the fact that LM rarely occurs in the splenic artery, splenic hilum. The risk factors for LM include CEA grading, LN status by CT, and T staging by CT. And we can use *Y* = -5.0 + *X*_1_ + 1.8*X*_3_ + 0.7*X*_4_ (*X*_1_, CEA grading, *X*_3_ = LN status by CT, *X*_4_ = T staging by CT, the critical value is 0.3) to estimate the possibility of LM, which has a better accuracy compared with CT.

## Background

Although the morbidity of gastric cancer (GC) is declining nowadays, it remains the second most common cause of cancer-related death worldwide.[1, 2, 3] The most important progress for gastric cancer in last century has been made of D2 lymphadenectomy, [4] which is the only treatment offering a cure hope. Thus, to obtain R0 resection, surgeons commonly perform this procedure even in cases of early gastric cancer (EGC). However, the uniform application of this highly invasive procedure carries significantly higher postoperative morbidity, mortality, and reoperation rates.[5]

It is well known that lymph node metastasis (LM) is an important prognostic risk factor.[6, 7, 8] And individualized treatment for GC patients requires selection reasonable therapeutic schedule according to patient’s specific *situation*; one important aspect is the evaluation for LM. LM in gastric cancer is nevertheless significantly complicated, with many related factors [9, 10], and there is still no good method to predict it.

With the development of medical technology, endoscopic mucosal resection (EMR) and endoscopic submucosal dissection (ESD) are being increasingly implemented in clinical settings. Although EMR or ESD can remove lesions in a minimally invasive manner, lymphadenectomy cannot be achieved by these techniques. Thus, it is very important to choose appropriate cases without LM.

There are currently many tests judging whether a GC patient has LM, such as endoscopic ultrasonography (EUS) or multislice spiral computed tomography (MSCT). According to the report of Feng XY et al, accuracy with regard to the presence of LM was 75.7% in EUS studies and 61.1% in MSCT studies.[11, 12] This level is clearly insufficient because an accurate determination of the LM status has an important effect on the choice of treatment. Regardless, there is no perfect examination or method to predict the possibility of LM.[13]

A sentinel node (SN) is defined as the first lymph node that receives lymphatic drainage from the primary tumor [14, 15], and a solitary metastatic lymph node could be regarded as an SN in gastric carcinoma [16].Due to the complexity of GC lymphatic drainage, sentinel lymph node biopsy has failed to achieve good results. Up to now, many studies have investigated the localization and distribution of SNs to provide useful information for sentinel lymph node biopsy. There are few studies involving the possibility of LM in GC. We speculate that compared to no lymph node metastasis (NLM), solitary lymph node metastasis (SLM) is a special state that from no lymph node metastasis to lymph node metastasis. And that means we can obtain these risk factors by comparison between these two groups. [17]

## Ethics Statement

The protocol was approved by the Sun Yat-sen University Cancer Center review board, in accordance with Chinese bioethical regulations. All patients provided written informed consent to offer related information in hospital.

## Patients and Methods

From July 2000 to July 2012, a retrospective analysis was performed of clinicopathologic data for GC patients at the Department of Gastric & Pancreatic Surgery, Sun Yat-sen University Cancer Center, State Key Laboratory of Oncology in South China. The criteria for inclusion in this study were as follows:

(1) No synchronization tumors;
(2) radical D2 lymphadenectomy was performed;
(3) Patient did not receive any neoadjuvant therapy, including chemotherapy, radiotherapy, and Chinese medicine;
(4) No recurrence cases;
(5) The number of clean lymph nodes was more than 14, with a metastatic lymph node number of 0 and 1;
(6) Complete follow-up data.

A total of 385 patients with gastric cancer were included. Among them were 303 patients (78.7%) with NLM and 82 patients (22.3%) with SLM, respectively. The follow-up visits ranged from 16 to 127 months; with an average of 52.3 ± 25.9 months (mean ± SD). The 5-year survival rates for NLM and SLM group was 86% and 70%, respectively. The average patient age (mean ± SD) was 56.88 ±11.3 years (ranging from 18 to 89 years), and more men than women (275 men versus 110 women) participated in the study. The carcinomas were located in the upper third of the stomach (U) in 124 patients (32.2%), the middle third (M) in 50 patients (13.0%), and the lower third (L) in 203 (52.7%) patients. There were also 3 patients with total gastric cancer and 5 patients with a tumor located in a residual anastomotic stomach. Distal-gastrectomy was performed in 236 patients, proximal-gastrectomy in 118 patients, total-gastrectomy in 26 patients, and total-residual stomach resection in 5 patients. The number of lymph nodes retrieved ranged from 14 to 67, with an average of 24.2 ± 9.2 (mean ± SD). Further data are presented in table 1.

**Table 1:** The clinicopathologic features between NLM group and SLM group.

**Parameters**	**NLM group N %**		**SLM group N %**		***P***
**Sex**					0.211
**Male**	213	70.3	62	75.6	
**Female**	90	29.7	20	24.4	
**Age**					0.807
**youth**	28	9.2	6	7.3	
**Middle-aged**	142	46.9	40	48.8	
**old-age**	133	43.9	36	43.9	
**CRP level**					0.142
**Normal**	264	87.1	67	81.7	
**Elevated**	39	12.9	15	18.3	
**CEA level**					0.028
**Normal**	268	88.4	65	79.3	
**Elevated**	35	11.6	17	20.7	
**CA-199 level**					0.066
**Normal**	281	92.7	71	86.6	
**Elevated**	22	7.3	11	13.4	
**CA-724 level**					0.064
**Normal**	257	84.4	63	76.8	
**Elevated**	46	15.5	19	23.2	
**Tumor size**					0.007
**≤3cm**	108	35.6	17	20.7	
**＞3cm**	195	64.4	65	79.3	
**Borrmann type**					0.073
**I**	33	10.9	6	7.3	
**II**	163	53.8	34	41.5	
**III**	99	32.7	39	47.6	
**IV**	8	2.6	3	3.7	
**Lauren type**					0.197
**inessential**	234	77.2	59	72.0	
**mixed**	69	22.8	23	28.0	
**Histological type**					0.080
**H**	22	7.3	0	0.0	
**M**	101	33.3	32	39.0	
**P**	145	47.9	39	47.6	
**S**	35	11.6	11	13.4	
T staging by CT*					0.000
**T1**	65	12.5	11	13.4	
**T2**	103	34.0	7	8.5	
**T3**	127	41.9	56	68.3	
**T4**	8	2.6	8	8.8	
LN status by CT**					0.000
**no enlargement of lymph nodes**	246	81.2	41	50.0	
**swollen lymph nodes**	57	18.8	41	50.0	

*.The T staging by CT was acquired after experienced image doctor read the pictures;

**.The LN status by CT was considered when the short-axis diameter was larger than 6 mm for perigastric lymph nodes and larger than 8 mm for the extraperigastric lymph nodes, especially nodes with arounded shape and enhancement on contrast-enhanced CT.

We simultaneously chose another group of GC patients from Sun Yat-sen University Gastrointestinal Hospital (SYSUGIH) to verify the LM risk model. The selection criteria were:

(1) No synchronization tumors;
(2) radical D2 lymphadenectomy was performed;
(3) Patient did not receive any neoadjuvant therapy, including chemotherapy, radiotherapy, and Chinese medicine;
(4) No recurrence cases;
(5) The number of clean lymph nodes was more than 14, and the pathological of LM diagnosis was complete.

There were a total of 210 GC patients. Among them were 73 patients (34.8%) with NLM and 137 patients (65.2%) with LM. The average patient age (mean ± SD) was 57.93± 13.32 years (ranging from 24 to 86 years). The follow-up visits ranged from 4 to 95, with an average of 43.2 ± 20.9 months (mean ± SD). Further data are presented in table 2.

**Table 2:** The clinicopathologic features in validation group.

**Clinical and pathological characteristics**	**Variable**
**Age (years)**	
Range	24–86
Median	57.93
**Gender**	
Male	142
Female	68
CEA level*	
Normal	172
Elevated	38
**T staging from CT**	
T1	30
T2	21
T3	112
T4	47
**LN status from CT**	
NO	88
Yes	122
Lymph node metastasis**	
NO	73
Yes	137

*.The scope of normal CEA is 0.00–5.00 ng/ml;

**.According the pathological to determine the presence of LM.

## Follow-Up

The follow-up way include outpatient follow-up, telephone follow-up, letter follow-up, short message platform follow-up and e-mail follow-up.

## Statistical Analysis

All the data were analyzed using the SPSS 13.0 statistics software. χ^2^ tests were used when appropriate to compare the distribution of individual variables between groups. A survival analysis was performed using the Kaplan–Meier method, and statistical comparisons of different factors were performed with the log-rank test. The COX model was used in a multivariate analysis, and a discriminate analysis was used to estimate the risk factors of LM. In all analyses, a two-tailed *P* value of 0.05 was considered statistically significant.

## Results

### Location and distribution of metastatic lymph nodes in gastric cancer

Among the 46 patients who had LM with a lower-third tumor, 34 (73.9%) had LM in the perigastric nodes (D1) close to the primary tumor, and No 3/6 was the most common site. The other 12 patients (26.1%) showed D2 station metastasis without D1 station involvement. Of the 5 patients with a middle-third tumor, 2 patients (40%) had LM in D1 station, whereas skip metastasis was found in 3 patients (60%). In 29 patients with an upper-third tumor, 19 patients (65.5%) had metastasis in D1 station, and skip metastasis occurred in 10 patients (34.5%).

In the D2 station, No. 12 was also involved, in addition to No 7, 8a, and 9. The detailed frequency of the different positions involved in D1 station and D2 station is provided in table 3.

**Table 3:** The location and distribution of metastatic lymph nodes in gastric cancer.

**Group**	**U (%)**	**M (%)**	**L (%)**
**No.1**	24.1		2.2
**No.2**	17.2	20.0	
**No.3**	20.7		32.6
**No.4**	3.4	20.0	4.3
**No.5**		20.0	17.4
**No.6**			19.6
**No.7**	34.5	40.0	15.2
**No.8**			6.5
**No.12**			2.2
**Compartment**			
**D1**	65.5	40.0	73.9
**D2**	34.5	60.0	26.1

### No significant survival difference between the skip LM patients and the other SLM patients

**Figure 1:**
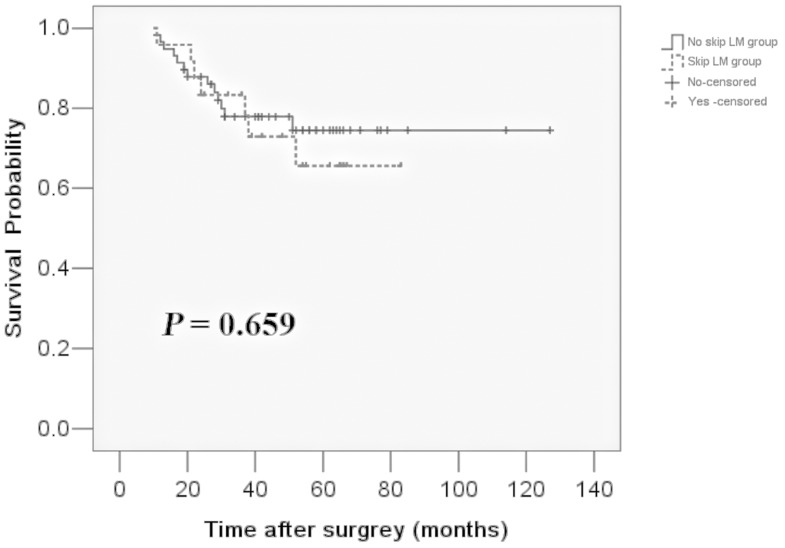
The 5-year survival of the groups between the skip LM patients and the other SLM patients is 65.6% and 74.5, respectively. The log-rank test revealed there was no significant difference between the two curves (χ^2^ = 0.195, *P* = 0.659).

Among the 82 patients who had SLM, 24 (29%) showed skip LM. The clinicopathologic features between the skip LM group and the other LM group is shown in table 4. Due to the small sample size, only a pathological T staging difference was found between the two groups. However, the logistic regression did not reveal statistical significance (*P* = 0.079), and the K-M analysis showed no survival difference between the two groups (*P* = 0.659). The survival curves are presented in Fig. 1.

**Table 4:** The clinicopathologic features between no skip SLM and skip SLM.

**Parameters**	**NLM group**		**SLM group**		***P***
	**N**	**%**	**N**	**%**	
**Sex**					0.175
Male	46	79.3	16	66.7	
Female	12	20.7	8	33.3	
**Age**					0.443
youth	5	8.6	1	4.2	
Middle-aged	30	51.7	10	41.7	
old-age	23	39.7	13	54.2	
**T staging from**					0.031
T1	10	17.2	1	4.2	
T2	7	12.1	0	0.0	
T3	34	58.6	22	91.7	
T4	7	12.1	1	4.2	
**Tumor size**					0.397
≤3cm	13	22.4	4	16.7	
>3cm	45	77.6	20	83.3	
**Tumor location**					0.653
U	19	33.9	10	41.7	
M	3	46.6	2	8.3	
L	34	15.5	12	50.0	
**Histological**					0.685
H	0	0.0	0	0.0	
M	22	37.9	10	41.7	
P	27	46.6	12	50.0	
S	9	15.5	2	8.3	
**Borrmann type**					0.630
I	4	6.9	2	8.3	
II	25	43.1	9	37.5	
III	26	44.8	13	54.2	
IV	346	5.2	0	0.0	
**Lauren type**					0.457
inessential	41	70.7	18	75.0	
mixed	17	29.3	6	25.0	
**Vascular invasion**					0.285
Y	37	63.8	13	53.2	
N	21	36.2	11	45.8	
**CRP level**					0.181
Normal	49	84.5	18	75.0	
Elevated	9	15.5	6	25.0	
**CEA level**					0.603
Normal	46	79.3	19	79.2	
Elevated	12	20.7	5	20.8	
**CA-199 level**					0.593
Normal	50	86.2	21	87.5	
Elevated	8	13.8	3	12.5	
**CA-724 level**					0.457
Normal	45	77.6	21	87.5	
Elevated	13	22.4	3	12.5	

### Univariate and multivariate analyses of prognosis

According to the K-M analysis, we found that the LM number, pathological T staging, Borrmann type, vascular invasion, and CRP grading were risk factors.

Furthermore, we used the COX regression model to analyze these risk factors to identify independent risk factors. The results showed that the LM number and pathological T staging were independent prognostic risk factors, whereas the other factors were excluded. All these results are shown in table 5.

**Table 5:** The univariate and multivariate analyses of overall survival in these patients.

	**univariate analyses**			**mulvariate analyses**		
	**HR**	**95%CI**	***P***	**HR**	**95%CI**	***P***
**Borrmann type**	1.581	1.094–2.285	0.015			
**vascular invasion**	1.675	1.013–2.770	0.044			
**CRP grading**	1.850	1.030–3.323	0.039			
**The LM**	2.165	1.264–3.709	0.005	1.784	1.027–3.097	0.040
**Pathological T staging**	1.774	1.268–2.481	0.001	1.665	1.177–2.327	0.004

### The LM risk in GC patients evaluated by discriminate analysis

The clinicopathologic features between the NLM and SLM groups are shown in table 1, which reveals differences in CEA level, tumor size, T staging by CT, and LN status by CT by correlation analysis.

We then obtained an unstandardized canonical equation by the discriminate analysis to ascertain whether GC patients have LM：*Y* = -5.350 + 0.928*X*_1_ + 0.336*X*_2_ + 1.769*X*_3_ + 0.648*X*_4_ (*X*_1_, CEA grading, *X*_2_, the size of the tumor, *X*_3_ = LN status by CT, *X*_4_ = T staging by CT). The values for all parameters are shown in table 6.

**Table 6:** The values of all parameters of the unstandardized canonical equation.

**Parameters**	**Values**
***X*_1_: the CEA grading**	
Normal	1
High	2
***X*_2_: the size of tumor**	
＜3cm	1
≥3cm	2
***X*_3_: LN status from CT**	
No enlargement of lymph nodes	1
Swollen lymph nodes	2
***X*_4_: T staging from CT**	
T1	1
T2	2
T3	3
T4	4

The two average discriminant values ($\bar{Y}$_*1*_ and $\bar{Y}$_*2*_) were obtained when the means of all independent variables ($\bar{X}$_*J*_) were put into the above equation. The critical discriminant value can be calculated by the following equation: $\bar{Y}$_*C*_ = ($\bar{Y}$_*1*_ + $\bar{Y}$_*2*_)/2. If *Y* of a case is larger than $\bar{Y}$_*C*_, it belongs to the group of LM; otherwise, it belongs to the NLM group. The critical value is 0.293.

Furthermore, we used the stepwise method to further refine and develop the equation *Y* = -4.990 + 0.973*X*_1_ + 1.800*X*_3_ + 0.696*X*_4_, for which the critical value is 0.290. In other words, if *Y* of a case is larger than 0.290, it belongs to the LM group; otherwise, it belongs to the NLM group. We can simplify the above formula to *Y* = -5.0 + *X*_1_ + 1.8*X*_3_ + 0.7*X*_4_ (the critical value is 0.3).

### Validation of the LM risk model using another group of GC patients from SYSUGIH

According to the literature, the accuracy of predicting the presence of LM was 75.7% in EUS studies and 61.1% in MSCT studies.[11] When we tested the accuracy of the equation *Y* = -5.0 + *X*_1_ + 1.8*X*_3_ + 0.7*X*_4_ (the critical value is 0.3), the authentication value ranged from 3.38 to-1.50, with an average of 1.00 ± 1.39. Among the values, there were 67 of less than 0.3, 143 of more than 0.3; 85.7% of cases that were correctly classified. The subgroup analysis showed accuracy for the NLM group of 75.3%, with 91.2% for the SLM group (table 7).

**Table 7:** The discriminant value of validation group.

	**<0.3**	**≥0.3**	***P***
No LM group*	55	18	0.00
**LM group**	12	125	

*: According to the results of pathological examination.

## Discussion

Gastric cancer is an important health problem, particularly in Asia, which has a high prevalence. Gastric cancer, even EGC, easily metastasizes to lymph nodes. The reports of the histopathological features of more than 13,000 patients, mainly Japanese, with EGC established that only 2% (range 0–4.8%) of patients with mucosal cancer have positive lymph nodes. When the tumor invades the submucosal layer (T1sm), this rates increases to approximately 20% (range 15–25%).[18] In order to get cured, D2 lymphadenectomy is employed even in the early gastric cancer.[19, 20] This choice is not appropriate, as higher morbidity and mortality rates are observed after D2 lymphadenectomy, compared to D1 resection. We think it is necessary to perform a D2 lymphadenectomy when the patient has LM. Whereas the patients who have little possibility of LM, we can narrow the scope of operation appropriately.[21, 22] The key is the assessment of LM.

In our research, differences of prognosis were found between NLM group and SLM group (*P* = 0.004). Further analysis showed that SLM is an independent risk factor. Other risk factors that affecting prognosis include the Borrmann type, vascular invasion, CRP grading, and pathological T staging. However, only SLM and pathological T staging were independent risk factors according to the COX model. It is possible that other factors have an indirect positive effect on survival through association with other cofactors. It is also possible that our sample size was insufficient.

Skip metastasis means SLM appears directly in the second station, and the reasons for skip metastasis remain unclear. The possible reasons include following: (1) occult metastases may remain hidden by routine histopathological examination; (2) there may be lymphatic routes that can drain directly to a second station; (3) the original site is too large, causing lymphatic drainage channel congestion and resulting in skip metastasis; (4) according to the theory of seed and soil, some first station lymph node microenvironments may not be suitable for tumor cell growth, resulting in skip metastasis.[23] According to report, skip metastasis rates of approximately 14% to 29% exist in SLM [7]; of these, there were 82 cases of gastric cancer with SLM and 24 cases of skip metastases, with an incidence of 29%. Therefore, skip metastases are notable, and rational lymphadenectomy should be performed on the SLM group. Due to the small number of cases, we didn’t find factor related to skip metastasis. There was also no difference in prognosis between no skip SLM group and skip SLM group, consistent with other studies [24]. For the reason of SLM rarely occurring in splenic artery, splenic hilum; it is inappropriate for the patient with SLM to do a D2 lymph node dissection.

Currently, an increasing number of patients with EGC are treated with endoscopic mucosal resection (EMR) or endoscopic submucosal dissection (ESD). The key to choose EMR or ESD is without LM. Unfortunately, diagnostic imaging techniques, including computed tomography and endosonography, are still unsatisfactory and do not provide a sufficiently accurate prediction of LM status in gastric cancer. Meantime, sentinel lymph node biopsy in gastric cancer also fails to obtain a good result due to the complexity of gastric cancer lymphatic drainage. Thus, we attempted to comprehensively use all available clinical data to predict the possibility of LM, and we believe SLM is a special group that reflects the critical state of LM. This state is from NLM to LM, and includes the risk factors leading to LM. As a result, we can find these risk factors through compare between NLM group and SLM group, resulting in a more accurate assessment to the status of LM.

According to discriminate analysis, we developed the unstandardized canonical equation *Y* = -5.0 + *X*_1_ + 1.8*X*_3_ + 0.7*X*_4_ (*X*_1_ = CEA grading, *X*_3_ = LN status by CT, *X*_4_ = T staging by CT, (the critical value is 0.3). This equation has a better accuracy compared with CT and EUS. While the lack of relevant data of EUS in the validation group and CT has a strong correlation with EUS at the same time, we finally decided to adopt CT to evaluate the status of LM. It maybe EUS has a better accuracy, and this will be employed in our future work.

The subgroup analysis showed a poor accuracy for the NLM group, which may be related to the evaluation standard of a positive lymph node. For example, an inflammatory hyperplasia lymph node leads to a false positive. The accuracy was higher in the LM group, showing that the positive result was relatively reliable. Of course, this is a retrospective study of small sample, and bias exists. Thus, further research is needed to determine the accuracy in our daily work. We also look forward to a better method for assessing the LM condition and guiding our treatment.

## Conclusions

SLM is an independent risk factor in gastric cancer.

There was no survival difference between the skip metastasis group and the other SLM group (*P* = 0.659)

It is inappropriate for the patient with SLM doing a standard D2 lymph node dissection, due to the LM rarely occurs in the splenic artery, splenic hilum.

The risk factors of LM included CEA grading, LN status by CT, T staging by CT. And we can use *Y* = -5.0 + *X*_1_ + 1.8*X*_3_ + 0.7*X*_4_ (*X*_1_, CEA grading, *X*_3_ = LN status by CT, *X*_4_ = T staging by CT, the critical value is 0.3) to estimate the possibility of LM, which has a better accuracy, especially when the results are positive.

## References

[1] Chen W, Zheng R, Zhang S, Zhao P, Li G, et al (2013) The incidences and mortalities of major cancers in China, 2009. Chin J Cancer 32: 106–112. 10.5732/cjc.013.10018 23452393 PMC3845591

[2] Jemal A, Siegel R, Ward E, Hao Y, Xu J, et al (2008) Cancer statistics, 2008. CA Cancer J Clin 58: 71–96. 10.3322/CA.2007.0010 18287387

[3] Li XP, Cao GW, Sun Q, Yang C, Yan B, et al (2013) Cancer incidence and patient survival rates among the residents in the Pudong New Area of Shanghai between 2002 and 2006. Chin J Cancer 32: 512–519. 10.5732/cjc.012.10200 23149312 PMC3845567

[4] Kitagawa Y, Takeuchi H, Takagi Y, Natsugoe S, Terashima M, et al (2013) Sentinel node mapping for gastric cancer: a prospective multicenter trial in Japan. J Clin Oncol 31: 3704–3710. 10.1200/JCO.2013.50.3789 24019550

[5] Aikou T, Kitagawa Y, Kitajima M, Uenosono Y, Bilchik AJ, et al (2006) Sentinel lymph node mapping with GI cancer. Cancer Metastasis Rev 25: 269–277. 10.1007/s10555-006-8507-3 16770539

[6] Huang B, Wang Z, Sun Z, Zhao B, Xu H (2011) A novel insight of sentinel lymph node concept based on 1–3 positive nodes in patients with pT1–2 gastric cancer. BMC Cancer 11: 18 10.1186/1471-2407-11-18 21241483 PMC3031262

[7] Liu CG, Lu P, Lu Y, Jin F, Xu HM, et al (2007) Distribution of solitary lymph nodes in primary gastric cancer: a retrospective study and clinical implications. World J Gastroenterol 13: 4776–4780. 17729400 10.3748/wjg.v13.i35.4776 PMC4611200

[8] Hirasawa T, Gotoda T, Miyata S, Kato Y, Shimoda T, et al (2009) Incidence of lymph node metastasis and the feasibility of endoscopic resection for undifferentiated-type early gastric cancer. Gastric Cancer 12: 148–152. 10.1007/s10120-009-0515-x 19890694

[9] Matsumoto M, Natsugoe S, Ishigami S, Uenosono Y, Takao S, et al (2003) Rapid immunohistochemical detection of lymph node micrometastasis during operation for upper gastrointestinal carcinoma. Br J Surg 90: 563–566. 10.1002/bjs.4083 12734863

[10] Cozzaglio L, Bottura R, Di Rocco M, Gennari L, Doci R (2011) Sentinel lymph node biopsy in gastric cancer: possible applications and limits. Eur J Surg Oncol 37: 55–59. 10.1016/j.ejso.2010.10.012 21115231

[11] Feng XY, Wang W, Luo GY, Wu J, Zhou ZW, et al (2013) Comparison of endoscopic ultrasonography and multislice spiral computed tomography for the preoperative staging of gastric cancer—results of a single institution study of 610 chinese patients. PLoS One 8: e78846 10.1371/journal.pone.0078846 24223855 PMC3815220

[12] Ahn HS, Lee HJ, Yoo MW, Kim SG, Im JP, et al (2009) Diagnostic accuracy of T and N stages with endoscopy, stomach protocol CT, and endoscopic ultrasonography in early gastric cancer. J Surg Oncol 99: 20–27. 10.1002/jso.21170 18937292

[13] Moenig SP, Luebke T, Baldus SE, Schroeder W, Bollschweiler E, et al (2005) Feasibility of sentinel node concept in gastric carcinoma: clinicopathological analysis of gastric cancer with solitary lymph node metastases. Anticancer Res 25: 1349–1352. 15865090

[14] Kitagawa Y, Kitajima M (2002) Gastrointestinal cancer and sentinel node navigation surgery. J Surg Oncol 79: 188–193. 10.1002/jso.10065.abs 11870670

[15] Tokunaga M, Ohyama S, Hiki N, Fukunaga T, Yamada K, et al (2009) Investigation of the lymphatic stream of the stomach in gastric cancer with solitary lymph node metastasis. World J Surg 33: 1235–1239. 10.1007/s00268-009-9985-6 19288280

[16] Gretschel S, Bembenek A, Ulmer C, Hunerbein M, Markwardt J, et al (2005) Prediction of gastric cancer lymph node status by sentinel lymph node biopsy and the Maruyama computer model. Eur J Surg Oncol 31: 393–400. 10.1016/j.ejso.2004.11.014 15837046

[17] Griniatsos J, Yiannakopoulou E, Gakiopoulou H, Alexandrou A, Dimitriou N, et al (2011) Clinical implications of the histologically and immunohistochemically detected solitary lymph node metastases in gastric cancer. Scand J Surg 100: 174–180. 22108745 10.1177/145749691110000307

[18] Roukos DH (2000) Current status and future perspectives in gastric cancer management. Cancer Treat Rev 26: 243–255. 10.1053/ctrv.2000.0164 10913380

[19] Kitagawa Y, Fujii H, Kumai K, Kubota T, Otani Y, et al (2005) Recent advances in sentinel node navigation for gastric cancer: a paradigm shift of surgical management. J Surg Oncol 90: 147–151, 151–152. 10.1002/jso.20220 15895450

[20] Tsujitani S, Oka S, Saito H, Kondo A, Ikeguchi M, et al (1999) Less invasive surgery for early gastric cancer based on the low probability of lymph node metastasis. Surgery 125: 148–154. 10.1016/S0039-6060(99)70258-8 10026747

[21] Ono H, Kondo H, Gotoda T, Shirao K, Yamaguchi H, et al (2001) Endoscopic mucosal resection for treatment of early gastric cancer. Gut 48: 225–229. 10.1136/gut.48.2.225 11156645 PMC1728193

[22] Ohgami M, Otani Y, Kumai K, Kubota T, Kim YI, et al (1999) Curative laparoscopic surgery for early gastric cancer: five years experience. World J Surg 23: 187–192, 192–193. 10.1007/PL00013167 9880430

[23] Lee SE, Lee JH, Ryu KW, Cho SJ, Lee JY, et al (2009) Sentinel node mapping and skip metastases in patients with early gastric cancer. Ann Surg Oncol 16: 603–608. 10.1245/s10434-008-0283-6 19127361

[24] Park SS, Ryu JS, Min BW, Kim WB, Kim SJ, et al (2005) Impact of skip metastasis in gastric cancer. ANZ J Surg 75: 645–649. 10.1111/j.1445-2197.2005.03485.x 16076325

